# Identifying Key Predictors and Developing a Machine Learning Model for Nurse Burnout in China

**DOI:** 10.1155/jonm/8179894

**Published:** 2026-07-13

**Authors:** Zirong Li, Qinghua Fan, Xiao Gan, Jinbi Wei, Yuhui Chen, Yanping Ying

**Affiliations:** ^1^ Department of Nursing, The First Affiliated Hospital of Guangxi Medical University, Nanning, Guangxi, China, gxmu.edu.cn; ^2^ Public Health College of Guangxi Medical University, Nanning, Guangxi, China; ^3^ Guangxi Nursing Quality Control Center, Nanning, Guangxi, China

**Keywords:** job burnout, machine learning, risk factor

## Abstract

**Background:**

Increasing work pressure has elevated burnout risk among Chinese nurses; identifying associated factors and developing predictive models are essential for early intervention.

**Aims:**

This study aims to develop a nurse burnout prediction model based on machine learning algorithms in order to assist nursing management in the future.

**Study Design:**

A cross‐sectional online survey of 1391 Chinese nurses (burnout rate: 75.7%) was conducted from June to September 2025 using snowball sampling. Burnout was measured by MBI‐GS, and 25 candidate variables were collected. Data were split 80/20 into training/validation sets. After variable selection (Boruta ∩ Group LASSO), seven algorithms were compared. Model evaluation used AUC, PR‐AUC, calibration, DCA, and SHAP. The optimal threshold (Youden index) defined three risk tiers, and a Shiny web calculator was developed.

**Results:**

Random forest achieved the best test performance (AUC = 0.879, PR‐AUC = 0.940, and sensitivity = 0.870). Key features included colleague relationship, work duration, exercise frequency, and night shift hours. SHAP analysis showed that harmonious colleague relationships, longer work duration, and regular exercise were associated with lower predicted risk, whereas longer night shifts and frequent training/exams were associated with higher predicted risk. Model calibration (HL *p* = 0.3505) and DCA confirmed clinical benefit. A Shiny web calculator was developed.

**Conclusion:**

The random forest model effectively predicts nurse job burnout with good discrimination and calibration. The Shiny web calculator developed based on this model provides a practical tool for early identification and stratified intervention in nursing management. External validation is needed to further confirm generalizability.

**Relevance to Clinical Practice:**

The predictive model can be integrated into hospital human resource and nursing information systems to enable automatic assessment of burnout risk. This facilitates early identification of high‐risk nurses, allowing managers to implement timely interventions such as adjusted schedules and psychological support. The deployed online calculator provides an accessible tool for dynamic monitoring and proactive management, supporting data‐driven decision‐making to improve nursing workforce stability and healthcare service quality.

**Permission to Reproduce Material From Other Sources:**

No materials from other sources were used in this manuscript that require permission.

**Reporting Guidelines:**

This study followed the TRIPOD + AI statement. The completed TRIPOD + AI checklist is provided as supporting information.

**Summary**
• What is known about the topic:
1. Compared with traditional regression algorithms, machine learning models perform better in handling high‐dimensional and complex data related to nurse burnout.
2. These models can effectively identify and analyze various risk factors, thereby predicting the phenomenon of burnout.
• What this paper adds:
1. By focusing the research on frontline clinical nurses, this study has provided new insights and ideas for preventing nurse burnout.
2. This study developed a random forest model that outperformed traditional methods; SHAP analysis explained the results, and a web calculator was constructed.

## 1. Introduction

Job burnout refers to a state of physical and mental fatigue and exhaustion experienced by individuals under prolonged work‐related stress, typically accompanied by emotional, attitudinal, and behavioral signs of depletion [1, 2]. Due to the nature of their profession, nurses are more susceptible to job burnout compared to other occupational groups. This phenomenon not only leads to sustained physical fatigue but also increases the risk of anxiety and depression, resulting in decreased work efficiency, a higher incidence of errors, and diminished professional identity, ultimately undermining nursing performance and posing risks to patient safety [3].

During the COVID‐19 pandemic, some inpatient medical demands were suppressed. With the conclusion of the pandemic, healthcare institutions have resumed normal operations, gradually releasing the pent‐up demand for hospitalizations, which has correspondingly increased operational pressures on hospitals [4, 5]. Concurrently, China’s new round of healthcare reform—specifically the Sanming Healthcare Reform—has been progressively implemented [6]. Against this backdrop, the work pressure faced by Chinese nurses is notably greater than that of their counterparts in other countries; thus, the prevention of job burnout is particularly critical [7–11]. The key to effectively preventing job burnout lies in identifying its associated influencing factors and predicting the likelihood of its occurrence.

## 2. Justification for Study

Currently, the construction of predictive models for job burnout has primarily relied on traditional statistical methods such as logistic regression, with insufficient exploration of modern machine learning techniques [12, 13]. Therefore, this study aims to employ machine learning methods to delve into the underlying patterns of nurse job burnout and, based on these findings, construct a predictive model. The goal is to provide reference for nursing managers to adjust management strategies, optimize human resource allocation, and thereby potentially address nurse job burnout, support better working conditions, and foster professional identity.

## 3. Aim and Objectives of Study

This study aims to develop a nurse burnout prediction model using machine learning algorithms in order to assist nursing management in the future.

## 4. Design and Methods

### 4.1. Data Collection Tools and Methods

From June to September 2025, an online survey was conducted using a snowball sampling method. Anonymized questionnaires were collected through the online survey platform, Wenjuanxing. Initially, 46 nurses from the First Affiliated Hospital of Guangxi Medical University were invited to participate and respond to the online questionnaire. Subsequently, these 46 participants shared the questionnaire link and QR code within their WeChat groups, inviting their classmates or colleagues to take part in the study. Later respondents were also encouraged to share the research further.

The Maslach Burnout Inventory‐General Survey (MBI‐GS) was employed to measure the outcomes of the study. The MBI‐GS, developed collaboratively by American psychologists Maslach and Jackson, consists of three dimensions: emotional exhaustion, cynicism, and inefficacy, comprising a total of 15 items [14–16]. The first two dimensions are scored positively, while the inefficacy dimension is scored negatively. Higher scores indicate more severe job burnout. This scale demonstrates good validity and reliability and is applicable to individuals aged 16 and above across various industries. In this study, the Chinese version of the MBI‐GS also showed excellent psychometric properties: the two‐factor structure explained 72.47% of the variance, factor loadings ranged from 0.68 to 0.914, KMO = 0.969, and Cronbach’s α = 0.941. The scoring method involves summing the scores from the three dimensions and dividing by 15 to obtain an average score. This average score is then multiplied by 20 to convert it into a standard score out of 100. A score below 50 indicates a favorable work status, whereas a score above 50 signifies the presence of job burnout. Based on this 50‐point cutoff, participants were divided into two groups for subsequent machine learning analysis: those with a standard score ≤ 50 were classified as the nonburnout group (favorable work status) and those with a score > 50 were classified as the burnout group (presence of job burnout). This binary grouping served as the dependent variable in the machine learning classification models.

Through a literature review, the risk factors for occupational burnout that were collected were determined, including the following three categories: demographic indicators, work‐related indicators, and health‐related indicators [5, 11, 15].

Demographic indicators include the following: gender, age_group, marital_status, maternity_status, education, and is_only_child.

Work‐related indicators include the following: job_title, work_duration_years, hospital_type (public, private), hospital_level (3A, 2A), department, weekly_work_hours, weekly_night_shift_hours, monthly_income, daily_patients_managed, family_support, medical_reform_feeling, patient_conflict, doctor_nurse_relationship, colleague_relationship, work_away_from_hometown, and monthly_training_frequency.

Health‐related indicators include the following: bmi_category, diet_regularity, and weekly_exercise_frequency.

### 4.2. Inclusion and Exclusion Criteria

Inclusion criteria were as follows: nurses working in comprehensive hospitals for more than 2 month. Exclusion criteria were as follows: (1) nurses currently on leave and (2) nurses not working in frontline clinical departments. Informed consent: The inclusion and exclusion criteria were presented at the top of the questionnaire, along with information regarding informed consent. Participants could proceed to fill out the questionnaire if they agreed to participate in the study; if they disagreed, they could opt not to complete the questionnaire and exit directly. This survey did not collect any identifiable information such as participants’ names, addresses, or workplaces.

### 4.3. Quality Control

IP address restriction: Each IP address is allowed to submit the questionnaire only once. Device restriction: Each device is permitted to complete the questionnaire only once. Logical checks: A logical check will be conducted on the questionnaires, and participants exhibiting logical inconsistencies will be excluded. For example, participants classified as youth with a work duration of 1 years holding a title of charge nurse or above will be removed from the study.

### 4.4. Data Analysis

#### 4.4.1. Statistical Methods

The data collected were analyzed using SPSS Version 23.0, with a significance level set at a two‐tailed threshold of *p* < 0.05 indicating statistical significance. Based on the MBI‐GS standard score cutoff of 50 (as described in the measurement section), participants were divided into two groups: the burnout group (score > 50) and the nonburnout group (score ≤ 50). All univariate group comparisons were performed between these two groups. The primary statistical methods employed are as follows: for normally distributed continuous data with equal variances, results are expressed as mean ± standard deviation and group comparison is conducted by using independent sample *t* test. For variables with nonnormal distribution, data are presented as median (interquartile range) and analyzed using the Mann–Whitney U test. Categorical variables are reported as frequency (percentage), with group comparisons conducted using the chi‐square test; when sample sizes are small, Fisher’s exact test is applied.

#### 4.4.2. Handling of Class Imbalance

To address the severe class imbalance (burnout prevalence: 75.7%, minority class: 24.3%), we did not adopt resampling techniques that may lose information (undersampling) or introduce synthetic noise (oversampling/SMOTE). Instead, we employed a more robust cost‐sensitive learning strategy using class‐weight adjustments. Specifically, we calculated inverse class weights based on class frequencies in the training set. For traditional machine learning models (e.g., logistic regression, random forest, Elastic Net, and K‐nearest neighbors [KNN]), we incorporated observation weights in the algorithm engine. For gradient boosting models (XGBoost and LightGBM), we set the scale_pos_weight parameter to the ratio of majority class to minority class samples. This cost‐sensitive strategy increases the penalty for misclassifying the minority class, thereby reducing the model’s bias toward predicting the majority class. The weighting was applied only to the training set; no test set information was used.

#### 4.4.3. Variable Selection Method

In medical research, variable selection is crucial for enhancing model efficiency, performance, and interpretability. To address multicollinearity and obtain a robust set of predictors, this study adopted a consensus‐based strategy combining two complementary methods. First, the Boruta algorithm—a random forest–based method that creates shadow features—identifies all relevant variables by comparing real features with randomized counterparts, ensuring that no potentially important predictor is overlooked. Second, Group LASSO regression applies L1 regularization to perform grouped variable selection, effectively mitigating multicollinearity and promoting sparsity. The final predictor set was defined as the intersection of variables selected by both approaches. This consensus ensures that only features consistently identified as important by two distinct methodologies are retained, yielding a parsimonious and reliable model [13, 17–19].

#### 4.4.4. Construction and Performance Evaluation of the Predictive Model

The R package caret was used to split the dataset into training (80%) and validation (20%) sets using the createDataPartition function. A 10‐fold cross‐validation strategy was employed on the training set to evaluate generalization ability.

Seven algorithms were compared: logistic regression, Elastic Net, KNN, random forest, AdaBoost, XGBoost, and LightGBM. For models including random forest and Elastic Net, hyperparameters were tuned using a combination of grid search and Bayesian optimization, with area under the ROC curve (AUC) as the objective function [20–23]. The optimization process started with 5 initial points followed by 10 iterations for global search.

Model performance was evaluated for both the training and validation sets using multiple metrics: AUC, precision–recall AUC (PR‐AUC) (more informative for imbalanced data), accuracy, sensitivity (recall), specificity, precision, F1‐score, and negative predictive value (NPV) [24].

To address severe class imbalance (75.7% burnout prevalence), we applied cost‐sensitive learning. For traditional models (logistic, Elastic Net, KNN, and random forest), observation weights (inverse of class frequencies) were used. For gradient boosting models (XGBoost and LightGBM), the scale_pos_weight parameter was set to the ratio of negative to positive samples [25].

After selecting the best model, SHapley Additive exPlanations (SHAP) analysis was performed to interpret the optimal model. We generated SHAP beeswarm plots, SHAP dependence plots, formalized interaction analysis plots, and interaction waterfall plots to visualize feature importance, direction of effects, and interaction patterns.

Calibration was assessed using the Hosmer–Lemeshow (HL) goodness‐of‐fit test, Spiegelhalter’s Z‐test, and the Brier score, and a calibration curve was plotted. Clinical utility was evaluated by decision curve analysis (DCA) for each model.

Finally, the optimal probability threshold was determined by maximizing the Youden index (sensitivity + specificity − 1). Based on this threshold, the study population was stratified into low‐, moderate‐, and high‐risk groups. The reclassification capability of this three‐tier system was visualized using Sankey diagrams and dual‐axis plots, demonstrating its practical value for nursing management.

### 4.5. Shiny Web Calculator Construction Method

Build a Shiny web calculator using the R software and deploy the application to the ShinyApps.io server using the Rsconnect package [26].

## 5. Ethics and Research Approval

This study was approved by the Ethics Committee of the First Affiliated Hospital of Guangxi Medical University (approval no. 2025‐E0820). The committee conducted a full board review and granted a waiver of signed informed consent based on the following justifications: (1) anonymous data collection—no personally identifiable information (e.g., names, addresses, and workplaces) was collected; (2) minimal risk—the survey involved only work‐related and health‐related questions, posing no more than minimal risk to participants; and (3) voluntary completion—the first page of the questionnaire contained a clear statement of study purpose, confidentiality, and the voluntary nature of participation, and returning a completed questionnaire was considered implied consent. The waiver was approved in accordance with Article 39 of the Chinese “Measures for Ethical Review of Biomedical Research Involving Human Subjects” and the Declaration of Helsinki (2013) Section 32. The study adhered to the ethical principles of the Declaration of Helsinki and relevant Chinese regulations.

## 6. Results

### 6.1. Basic Characteristic and Univariate Analysis

Finally, nurses from 25 provincial administrative regions in China participated in this study. In this study, a total of 1407 questionnaires were collected, among which 1391 were valid (see exclusion criteria in the Quality Control section). Out of the 1391 respondents, 1053 (75.7%) showed symptoms of job burnout. The analysis results showed that there were statistically significant differences in 16 variables between the two groups (*p* < 0.05, including continuous variables: work_duration_years, weekly_night_shift_hours, weekly_exercise_frequency, and monthly_training_frequency and categorical variables: age_group, maternity_status, job_title, hospital_level, marital_status, monthly_income, daily_patients_managed, family_support, doctor_nurse_relationship, patient_conflict, colleague_relationship, and is_only_child). Specifically, compared with the nonburnout group, the burnout group showed shorter work_duration_years, longer weekly_night_shift_hours, and lower weekly_exercise_frequency; in terms of demographics and working environment, the proportion of young, unmarried, nonpregnant, and only‐child individuals in the burnout group was higher, and the family_support level was lower, the doctor_nurse_relationship was less harmonious, and there were significant differences in monthly_income distribution and the daily_patients_managed. For further details, please refer to Tables 1 and 2.

**TABLE 1:** Cross (chi‐square) analysis results.

Characteristic	Classification	Result (%)		Overall	*χ* ^2^	*p*
		Positive	Negative	1391		
Gender	Female	963 (91.45)	298 (88.17)	1261 (90.65)	3.264	0.071
	Male	90 (8.55)	40 (11.83)	130 (9.35)		

Age group	Middle‐aged	310 (29.44)	76 (22.49)	386 (27.75)	298.599	0.000^∗∗^
	Senior	22 (2.09)	116 (34.32)	138 (9.92)		
	Youth	721 (68.47)	146 (43.20)	867 (62.33)		

BMI category	Normal	302 (28.68)	102 (30.18)	404 (29.04)	1.643	0.44
	Overweight	584 (55.46)	192 (56.80)	776 (55.79)		
	Underweight	167 (15.86)	44 (13.02)	211 (15.17)		

Maternity_status	Have children	457 (43.40)	214 (63.31)	671 (48.24)	40.637	0.000^∗∗^
	Childless	596 (56.60)	124 (36.69)	720 (51.76)		

Job title	Chief nurse	53 (5.03)	36 (10.65)	89 (6.40)	127.613	0.000^∗∗^
	Nurse‐in‐charge	241 (22.89)	48 (14.20)	289 (20.78)		
	Associate chief nurse	111 (10.54)	115 (34.02)	226 (16.25)		
	Nurse	377 (35.80)	80 (23.67)	457 (32.85)		
	Senior nurse	271 (25.74)	59 (17.46)	330 (23.72)		

Hospital level	Tertiary Grade A	915 (86.89)	332 (98.22)	1247 (89.65)	35.393	0.000^∗∗^
	Secondary Grade A	138 (13.11)	6 (1.78)	144 (10.35)		

Hospital type	Public	1026 (97.44)	334 (98.82)	1360 (97.77)	2.238	0.135
	Private	27 (2.56)	4 (1.18)	31 (2.23)		

Department	TCM	109 (10.35)	25 (7.40)	134 (9.63)	6.82	0.338
	Other	19 (1.80)	9 (2.66)	28 (2.01)		
	Internal Medicine	168 (15.95)	55 (16.27)	223 (16.03)		
	Surgery	209 (19.85)	72 (21.30)	281 (20.20)		
	Emergency	175 (16.62)	46 (13.61)	221 (15.89)		
	Operating Room	271 (25.74)	101 (29.88)	372 (26.74)		
	ICU	102 (9.69)	30 (8.88)	132 (9.49)		

Education	Technical secondary	15 (1.42)	9 (2.66)	24 (1.73)	3.748	0.441
	Doctor	3 (0.28)	0 (0.00)	3 (0.22)		
	Junior college	183 (17.38)	54 (15.98)	237 (17.04)		
	Bachelor	809 (76.83)	263 (77.81)	1072 (77.07)		
	Master	43 (4.08)	12 (3.55)	55 (3.95)		

Marital status	Widowed	8 (0.76)	5 (1.48)	13 (0.93)	38.78	0.000^∗∗^
	Married	417 (39.60)	179 (52.96)	596 (42.85)		
	Unmarried	566 (53.75)	118 (34.91)	684 (49.17)		
	Divorced	62 (5.89)	36 (10.65)	98 (7.05)		

Diet regularity	No	599 (56.89)	180 (53.25)	779 (56.00)	1.369	0.242
	Yes	454 (43.11)	158 (46.75)	612 (44.00)		

Monthly income (CNY)	0–2000	67 (6.36)	12 (3.55)	79 (5.68)	76.038	0.000^∗∗^
	2000–4000	166 (15.76)	11 (3.25)	177 (12.72)		
	4000–6000	460 (43.68)	138 (40.83)	598 (42.99)		
	6000–8000	257 (24.41)	96 (28.40)	353 (25.38)		
	> 8000	103 (9.78)	81 (23.96)	184 (13.23)		

Daily patients managed	0–10	456 (43.30)	224 (66.27)	680 (48.89)	69.772	0.000^∗∗^
	10–20	465 (44.16)	110 (32.54)	575 (41.34)		
	20–30	116 (11.02)	4 (1.18)	120 (8.63)		
	> 30	16 (1.52)	0 (0.00)	16 (1.15)		

Family support	Unsupportive	660 (62.68)	132 (39.05)	792 (56.94)	58.245	0.000^∗∗^
	Supportive	393 (37.32)	206 (60.95)	599 (43.06)		

Medical_reform_feeling	Negative	481 (45.68)	150 (44.38)	631 (45.36)	0.175	0.676
	Positive	572 (54.32)	188 (55.62)	760 (54.64)		

Doctor–nurse relationship	Not harmonious	629 (59.73)	149 (44.08)	778 (55.93)	25.429	0.000^∗∗^
	Harmonious	424 (40.27)	189 (55.92)	613 (44.07)		

Patient conflict history	No	523 (49.67)	189 (55.92)	712 (51.19)	4	0.046^∗^
	Yes	530 (50.33)	149 (44.08)	679 (48.81)		

Colleague relationship	Not harmonious	735 (69.80)	71 (21.01)	806 (57.94)	249.992	0.000^∗∗^
	Harmonious	318 (30.20)	267 (78.99)	585 (42.06)		

Is only child	No	255 (24.22)	146 (43.20)	401 (28.83)	44.919	0.000^∗∗^
	Yes	798 (75.78)	192 (56.80)	990 (71.17)		

Work away from hometown	No	341 (32.38)	121 (35.80)	462 (33.21)	1.345	0.246
	Yes	712 (67.62)	217 (64.20)	929 (66.79)		

Abbreviation: TCM = traditional Chinese medicine.

^∗^ *p* < 0.05.

^∗∗^ *p* < 0.01.

**TABLE 2:** Analysis results of continuous variables.

	Result mean ± standard deviation/(P_25_, P_75_)		*t*	*p*
	Positive (*n* = 1053)	Negative (*n* = 338)		
Work duration (years)	8.40 ± 8.94	18.40 ± 15.12	−11.525	0.000^∗∗^
Weekly work hours	43.30 ± 5.98	42.72 ± 5.75	1.601	0.11
Weekly night shift hours	12.10 ± 6.53	8.08 ± 7.11	9.636	0.000^∗∗^
Weekly exercise frequency	1.55 ± 1.90	2.52 ± 2.32	−7.006	0.000^∗∗^
Monthly training frequency	2.0 (2.0, 3.0)	2.0 (1.0, 3.0)	−5.557	0.000^∗∗^

^∗∗^ *p* < 0.01.

### 6.2. Variable Selection and Collinearity Diagnosis Results

**FIGURE 1:**
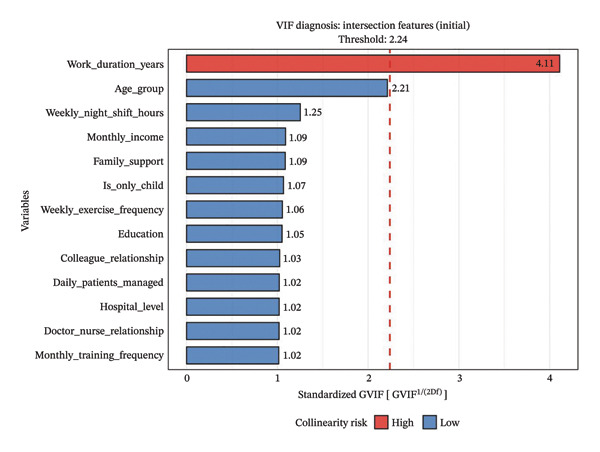
Initial VIF diagnosis for the 13 features selected by boruta ∩ group LASSO.

**FIGURE 2:**
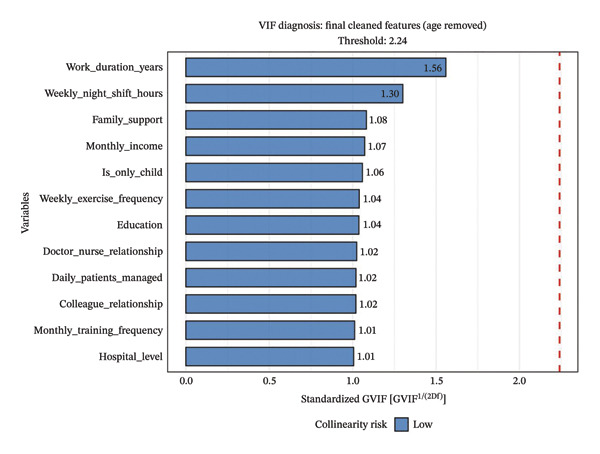
Final VIF diagnosis after removing age_group (12 features retained).

After obtaining the preliminary core feature set via the intersection of Boruta and Group LASSO, we performed a rigorous multicollinearity diagnosis using the variance inflation factor (VIF); for categorical variables with more than two levels, we calculated the generalized VIF (GVIF) and used its standardized form GVIF^(1/(2Df)) (threshold 2.24, equivalent to VIF = 5 for a continuous variable). As shown in Figure 1, the standardized GVIF for work_duration_years was 4.11, substantially exceeding the threshold, while all other variables (including age_group) were below 1.10, indicating that the high GVIF was driven by a strong linear relationship between work_duration_years and age_group. To resolve this issue while preserving maximal clinical information, we manually removed age_group (retaining the continuous work_duration_years), after which the standardized GVIF for work_duration_years dropped to 1.56 and all remaining GVIF values fell below 1.30 (Figure 2). The final set of 12 predictors entered into all subsequent models consisted of the following: work_duration_years, weekly_night_shift_hours, monthly_income, family_support, is_only_child, weekly_exercise_frequency, education, colleague_relationship, daily_patients_managed, hospital_level, doctor_nurse_relationship, and monthly_training_frequency.

### 6.3. Class Imbalance Handling Results

**FIGURE 3:**
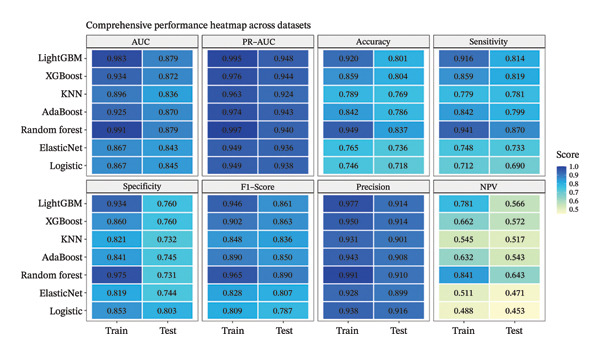
Performance heatmap of all models after class imbalance handling.

To address the severe class imbalance (75.7% burnout prevalence), we applied a cost‐sensitive learning strategy using class‐weight adjustments. As shown in the comprehensive performance heatmap (Figure 3), all seven models showed feasible performance on the test set, with sensitivity ranging from 0.690 to 0.870 and specificity ranging from 0.731 to 0.803. Random forest achieved the highest test sensitivity of 0.870, with a test specificity of 0.731. Tree‐based models achieved outstanding PR‐AUC, with random forest achieving a PR‐AUC of 0.940, showing strong robustness under imbalance. These results confirmed that cost‐sensitive weighting effectively alleviated majority‐class bias and enabled reliable identification of burnout cases.

### 6.4. Model Development and Validation Results

All seven candidate models were successfully trained on the 80% training set and validated on the 20% independent test set. All models demonstrated good performance on the training set (see Table 3 for detailed training metrics).

**TABLE 3:** Performance metrics of all seven models on the training set.

Evaluation index	AdaBoost	ElasticNet	KNN	LightGBM	Logistic	Random forest	XGBoost
AUC	0.925 [0.907–0.940]	0.867 [0.841–0.892]	0.896 [0.874–0.915]	0.983 [0.977–0.988]	0.867 [0.842–0.892]	0.991 [0.988–0.995]	0.934 [0.918–0.950]
PR‐AUC	0.974 [0.967–0.981]	0.949 [0.935–0.961]	0.963 [0.954–0.972]	0.995 [0.992–0.996]	0.949 [0.935–0.961]	0.997 [0.996–0.998]	0.976 [0.969–0.983]
Accuracy	0.842 [0.820–0.862]	0.765 [0.737–0.791]	0.789 [0.766–0.812]	0.920 [0.903–0.936]	0.746 [0.719–0.771]	0.949 [0.936–0.961]	0.859 [0.837–0.878]
Sensitivity	0.842 [0.816–0.866]	0.748 [0.717–0.778]	0.779 [0.752–0.806]	0.916 [0.896–0.933]	0.712 [0.683–0.739]	0.941 [0.925–0.955]	0.859 [0.835–0.881]
Specificity	0.841 [0.795–0.887]	0.819 [0.770–0.866]	0.821 [0.771–0.868]	0.934 [0.905–0.962]	0.853 [0.810–0.895]	0.975 [0.955–0.992]	0.860 [0.816–0.898]
F1‐score	0.890 [0.873–0.905]	0.828 [0.807–0.849]	0.848 [0.829–0.866]	0.946 [0.933–0.957]	0.809 [0.786–0.830]	0.965 [0.956–0.974]	0.902 [0.886–0.916]
Precision	0.943 [0.925–0.961]	0.928 [0.907–0.947]	0.931 [0.911–0.949]	0.977 [0.968–0.987]	0.938 [0.917–0.956]	0.991 [0.985–0.997]	0.950 [0.935–0.965]
NPV	0.632 [0.584–0.686]	0.511 [0.467–0.561]	0.545 [0.502–0.594]	0.781 [0.738–0.821]	0.488 [0.447–0.533]	0.841 [0.799–0.878]	0.662 [0.616–0.714]

On the test set (see Table 4 for detailed test metrics), random forest was the top‐performing model across most metrics. It achieved the highest AUC (0.879, 95% CI: 0.829–0.927)—tied with LightGBM—and the highest accuracy (0.837), sensitivity (0.870), F1‐score (0.890), and NPV (0.643). Notably, random forest also achieved an excellent PR‐AUC of 0.940 on the test set, which is substantially higher than the baseline prevalence of 75.7%, indicating strong robustness to class imbalance and reliable identification of the minority class (nonburnout nurses). Its sensitivity of 0.870 indicates that random forest correctly identified 87.0% of nurses who actually experienced burnout, a key advantage for identifying candidates for early intervention in clinical practice. LightGBM and XGBoost also yielded competitive test AUCs (0.879 and 0.872, respectively), but random forest consistently outperformed them in accuracy, sensitivity, and F1‐score. In contrast, logistic regression and Elastic Net had test AUCs around 0.845 and sensitivity around 0.69–0.73, which were substantially lower than those of random forest.

**TABLE 4:** Test set performance of the predictive models.

Evaluation index	AdaBoost	ElasticNet	KNN	LightGBM	Logistic	Random forest	XGBoost
AUC	0.870 [0.817–0.915]	0.843 [0.785–0.898]	0.836 [0.770–0.893]	0.879 [0.831–0.922]	0.845 [0.788–0.898]	0.879 [0.829–0.927]	0.872 [0.822–0.917]
PR‐AUC	0.943 [0.906–0.972]	0.936 [0.904–0.964]	0.924 [0.879–0.959]	0.948 [0.914–0.974]	0.938 [0.907–0.964]	0.940 [0.899–0.973]	0.944 [0.906–0.973]
Accuracy	0.786 [0.740–0.832]	0.736 [0.682–0.783]	0.769 [0.717–0.812]	0.801 [0.756–0.841]	0.718 [0.661–0.769]	0.837 [0.798–0.877]	0.804 [0.758–0.845]
Sensitivity	0.799 [0.746–0.850]	0.733 [0.672–0.789]	0.781 [0.722–0.834]	0.814 [0.758–0.863]	0.690 [0.625–0.751]	0.870 [0.826–0.913]	0.819 [0.762–0.866]
Specificity	0.745 [0.642–0.844]	0.744 [0.644–0.845]	0.732 [0.627–0.835]	0.760 [0.656–0.853]	0.803 [0.695–0.891]	0.731 [0.625–0.832]	0.760 [0.649–0.857]
F1‐score	0.850 [0.813–0.885]	0.807 [0.758–0.847]	0.836 [0.794–0.871]	0.861 [0.822–0.893]	0.787 [0.732–0.829]	0.890 [0.859–0.919]	0.863 [0.827–0.896]
Precision	0.908 [0.866–0.944]	0.899 [0.856–0.941]	0.901 [0.859–0.942]	0.914 [0.873–0.949]	0.916 [0.874–0.957]	0.910 [0.869–0.946]	0.914 [0.873–0.951]
NPV	0.543 [0.448–0.643]	0.471 [0.379–0.565]	0.517 [0.421–0.610]	0.566 [0.462–0.667]	0.453 [0.365–0.543]	0.643 [0.540–0.740]	0.572 [0.465–0.667]

Considering the independent test set results, random forest is selected as the optimal prediction model for nurse burnout. Its combination of high AUC, high sensitivity, balanced precision, and excellent PR‐AUC makes it particularly suitable for identifying at‐risk nurses in clinical settings, even under severe class imbalance.

**FIGURE 4:**
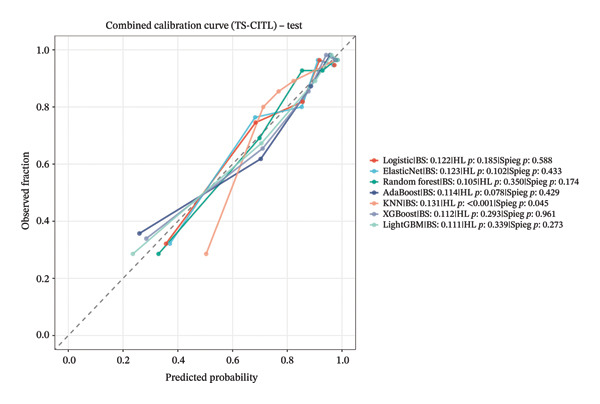
Combined calibration curves of the seven candidate models on the test set.

We further assessed the calibration of the optimal random forest model using the HL test with five quantile groups and Spiegelhalter’s Z‐test. On the test set, the Brier score was 0.1052, the HL test yielded a *p* value of 0.3505 (> 0.05), and the Spiegelhalter test gave a *p* value of 0.1735, indicating no statistically significant deviation between predicted probabilities and observed burnout rates. The calibration curve is presented in Figure 4.

**FIGURE 5:**
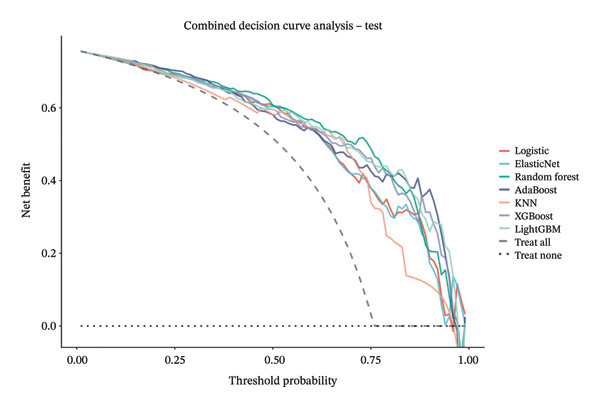
Combined decision curve analysis (DCA) of the seven machine learning models on the test set.

To evaluate clinical utility, we performed DCA on the test set (Figure 5). The DCA curve for random forest showed that across a wide range of threshold probabilities, it provided higher net benefit than both “treat‐all” and “treat‐none” strategies, confirming its potential value for real‐world nursing management.

The optimal probability threshold for identifying occupational burnout was determined as 0.527 using the maximum Youden index from the ROC curve in the test set. To enhance clinical applicability and support refined risk‐stratified interventions, three clinical risk grades were defined using this threshold and half its value (0.264) as cutoffs: low risk (< 0.264), moderate risk (0.264–0.527), and high risk (> 0.527).

**FIGURE 6:**
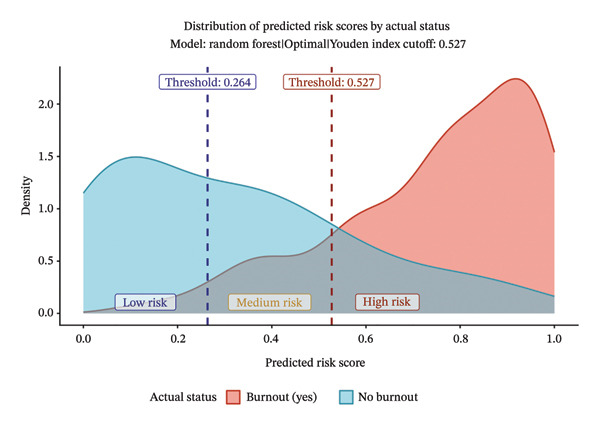
Probability density distribution of predicted risk scores for burnout and nonburnout nurses.

**FIGURE 7:**
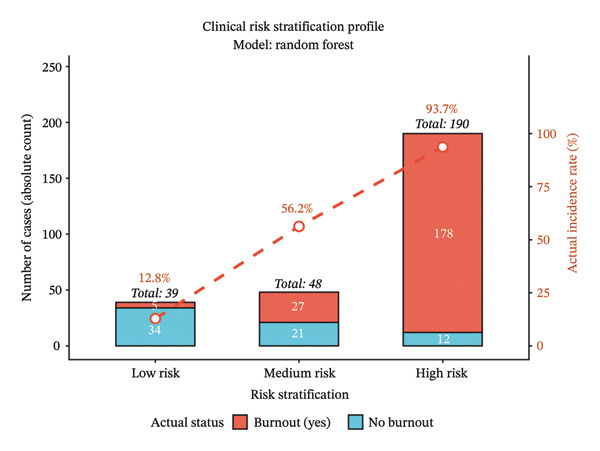
Dual‐axis plot showing risk‐stratified burnout incidence across low‐, moderate‐, and high‐risk groups.

As shown in the probability density distribution plot (Figure 6) of predicted risk scores in the test set, the random forest model achieved excellent separation between the two groups. Nurses with actual occupational burnout exhibited a pronounced right‐skewed distribution, with predicted scores concentrated mainly to the right of the high‐risk threshold (0.527). In contrast, scores for nurses without burnout were clustered predominantly to the left of the low‐risk threshold (0.264). The overlapping area of the two density curves fell precisely within the moderate‐risk interval (0.264–0.527), indicating satisfactory population stratification and clinical validity. Dual‐axis analysis (Figure 7) in the test set demonstrated a significant stepwise increase in actual burnout rates with ascending predicted risk levels: 12.8% in the low‐risk group, 56.2% in the moderate‐risk group, and 93.7% in the high‐risk group. The high‐risk category captured 84.8% of true burnout cases, confirming strong early‐warning performance in the test set.

**FIGURE 8:**
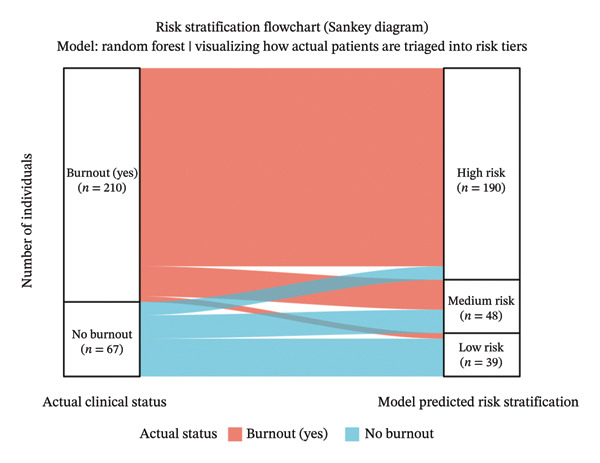
Sankey diagram demonstrating the model′s classification performance in risk stratification.

The Sankey diagram (Figure 8) and corresponding Table 5 further illustrated the classification performance in the test set. Among 210 nurses with confirmed burnout, 178 (84.8%) were correctly stratified into the high‐risk group, 27 (12.9%) into the moderate‐risk group, and only 5 (2.4%) were misclassified into the low‐risk group. Of 67 nurses without burnout, 34 (50.7%) were correctly assigned to the low‐risk group, 21 (31.3%) to the moderate‐risk group, and 12 (17.9%) were falsely classified into the high‐risk group.

**TABLE 5:** Risk stratification performance of the random forest model on the test set.

Risk_stratification	Total_patients	Burnout_cases	No_burnout_cases
Low risk	39	5	34
Medium risk	48	27	31
High risk	190	178	12

Collectively, the model successfully allocated 97.6% of true burnout cases to the moderate‐ and high‐risk intervention groups in the test set, while retaining more than half of nonburnout nurses in the low‐risk group. These results demonstrate robust stratification efficacy and reliable early‐warning ability, supporting the utility of this model for the early identification and precision intervention of occupational burnout.

**FIGURE 9:**
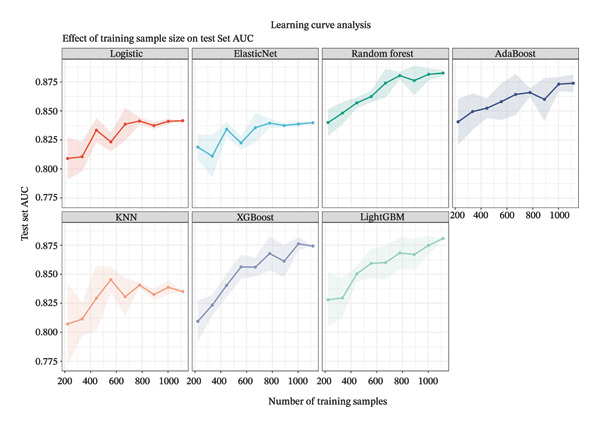
Learning curves of the seven candidate models.

Finally, we examined learning curves to assess sample size adequacy (Figure 9). The curve plateaued as the training sample size increased, demonstrating that the model has sufficiently learned the underlying data patterns without severe underfitting. This supports that our sample size (*n* = 1391) is adequate for the developed random forest model.

While further external validation is warranted, the current findings support random forest as a robust and clinically useful tool for predicting nurse burnout.

### 6.5. The Best Model SHAP Results

#### 6.5.1. The Magnitude and Directionality of the Key Feature Effect

**FIGURE 10:**
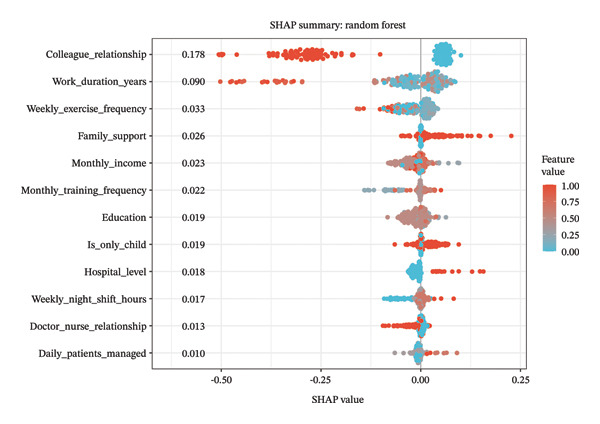
SHAP beeswarm plot of feature importance for the random forest model.

The SHAP beeswarm plot (Figure 10) revealed that the top ten most important features in our random forest model were, in descending order: colleague relationship (0.178), work duration years (0.090), weekly exercise frequency (0.033), family support (0.026), monthly income (0.023), monthly training frequency (0.022), education (0.019), being an only child (0.019), hospital level (0.018), and weekly night shift hours (0.017). In terms of directionality, harmonious colleague relationships, longer work duration, and higher exercise frequency exhibited strong “protective” patterns (negative SHAP values), meaning that higher values of these features were associated with lower predicted burnout risk. Conversely, weekly night shift hours was among the top ten features that showed a “risk‐promoting” effect, with longer night shifts being associated with higher predicted risk.

**FIGURE 11:**
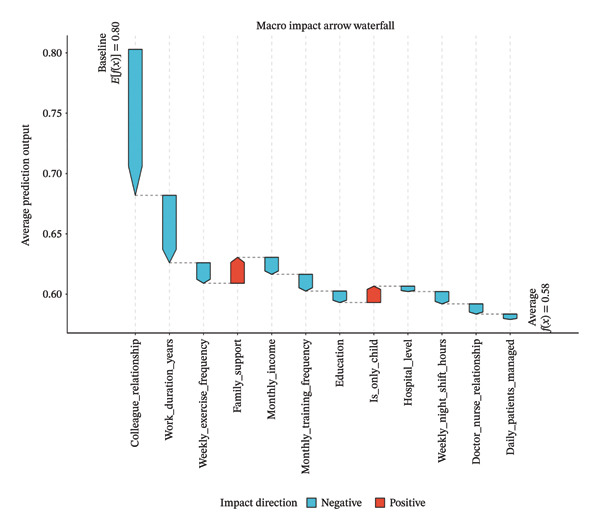
SHAP waterfall plot of the overall feature impact on the model′s average prediction.

**FIGURE 12:**
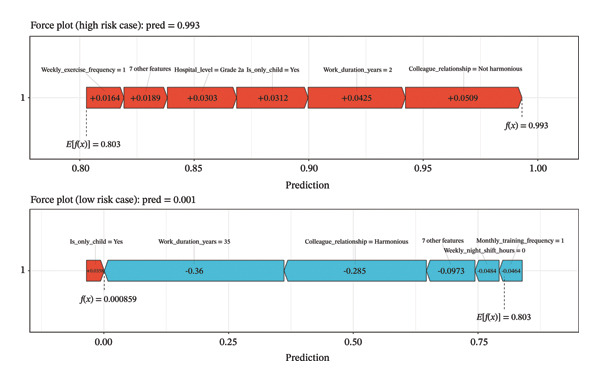
SHAP force plots for high‐risk and low‐risk individuals predicted by the random forest model.

Combining the overall impact waterfall plot (Figure 11) and SHAP force plots (Figure 12), the model’s features revealed a predominantly protective‐driven trend overall, while individual cases demonstrated a “double‐edged sword” effect of key features. Under the combined action of all features, multiple protective factors (blue negative arrows) worked together, pulling the model’s average predicted value from a baseline of 0.80 down to a final value of 0.58. Overall, the feature set exerted a predominantly risk‐lowering (protective) effect, with colleague relationship and work duration forming the core mechanism driving the decrease in predicted risk. In contrast, family support and being an only child showed a weak overall positive (risk‐increasing) contribution. From the force plot perspective, the decisive role of core features on individual outcomes was vividly illustrated. For a high‐risk individual (predicted probability: 0.993), “poor colleague relationship” (+0.0509) and “very short work duration (only 2 years)” (+0.0425) acted as risk factors (red arrows), jointly pushing the predicted risk from the baseline sharply upward to the peak. Conversely, for a low‐risk individual (predicted probability near the safety baseline of 0.001), “long work duration (35 years)” (pulling down by −0.360) and “harmonious colleague relationship” (pulling down by −0.285) brought the final prediction down to the safe level.

#### 6.5.2. Nonlinear Effect’s SHAP Local Dependence Graph

**FIGURE 13:**
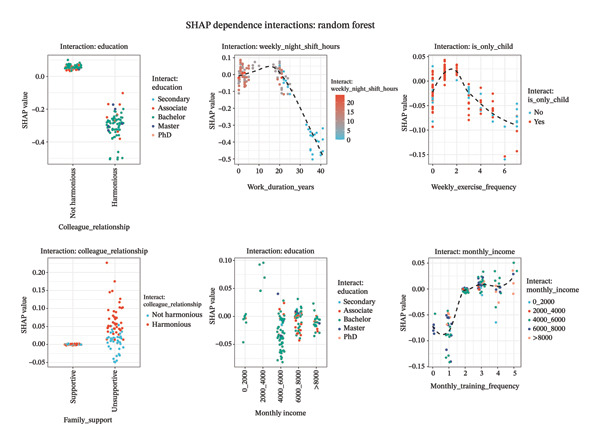
SHAP dependence plots showing nonlinear effects and synergistic interactions between key features.

We constructed SHAP dependence plots (Figure 13) and fitted LOESS smooth curves for continuous variables to characterize their nonlinear dynamic changes. Color mapping was used to visually reveal nonlinear synergistic effects between features. In the plot for colleague_relationship, SHAP values fell markedly into the negative range (indicating protective effects) when colleague relationship was harmonious. Data points representing high educational attainment (Master/PhD) were concentrated at the bottom of the cluster, with SHAP values reaching −0.3 to −0.4. This visually demonstrates that harmonious colleague relationships provide a fundamental risk‐reducing effect, while high educational background further strengthens and amplifies this protective impact.

The LOESS curve showed that SHAP values were generally positive when work duration was relatively short. Data points representing longer weekly night‐shift hours were located at the top of the plot, indicating that young work tenure plus long night shifts constituted the most detrimental combination for adverse outcomes. However, as work duration exceeded approximately 20 years, the curve declined steeply and remained in the negative range. Even with substantial night‐shift hours, corresponding SHAP values remained below zero, suggesting that longer work experience strongly buffers against the negative risks associated with night shifts.

In the plot for weekly_exercise_frequency, burnout risk decreased with increasing exercise frequency and plateaued at approximately 4–6 sessions per week. Interactive analysis further revealed that, within the optimal exercise range, points for participants who were not only children were lower than those for only children. This indicates that although increased exercise is universally beneficial, such interventions yield significantly stronger positive gains (reflected by larger negative SHAP values) among non‐only children.

In the interaction plot between monthly_income and education, clear vertical stratification was observed in the highest income group (> 8000). Participants with high education combined with high income reached the lowest SHAP values (most negative) across the entire plot. This indicates a significant synergistic interaction between high education and high income, which jointly and strongly enhances the risk‐reducing effect. Similar synergistic amplification effects were also confirmed between family support and colleague relationship, as well as between monthly training frequency and monthly income.

#### 6.5.3. Formalized Interaction Analysis

**FIGURE 14:**
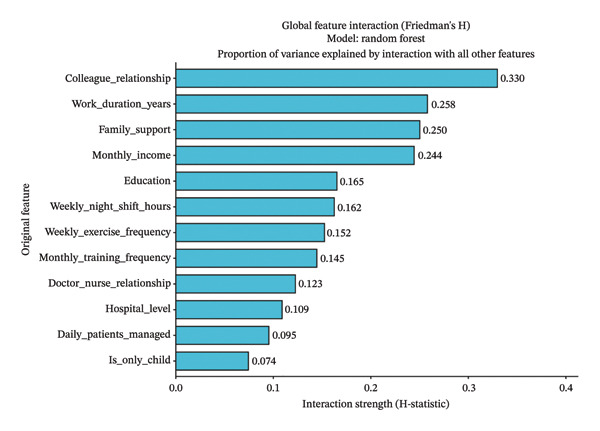
Global feature interaction strength (Friedman′s H‐statistic) of the random forest model.

**FIGURE 15:**
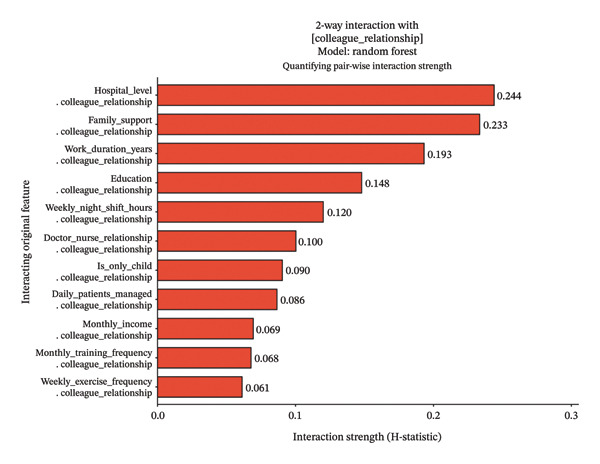
Pairwise interaction strength of colleague_relationship with other features (Friedman′s H‐statistic) in the random forest model.

**FIGURE 16:**
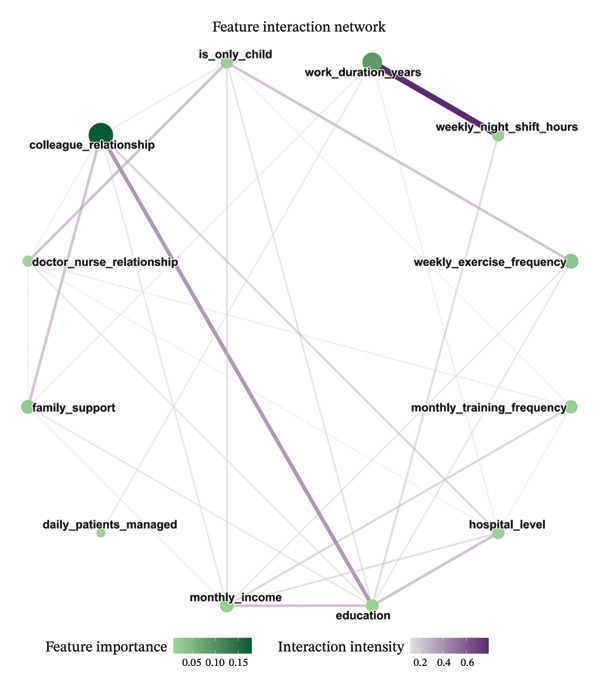
Feature interaction network visualizing the strength of interfeature associations in the random forest model.

Friedman’s H‐statistic was applied to systematically quantify global feature interaction strength and two‐way interaction effects to reveal the proportion of model prediction variance explained by feature interactions. Global interaction analysis (Figure 14) demonstrated that colleague_relationship presented the highest global interaction strength (*H* = 0.330), serving as the most critical interaction hub in the model. Two‐way interaction analysis (Figure 15) further confirmed that colleague_relationship exhibited the strongest pairwise synergistic effects with hospital_level (*H* = 0.244) and family_support (*H* = 0.233), providing rigorous statistical support for the interaction patterns observed in the preceding SHAP dependence plots. The feature interaction network (Figure 16) intuitively visualized the intensity of inter‐feature associations; it not only validated the results of the H‐statistic but also highlighted a strong interactive linkage between work_duration_years and weekly_night_shift_hours, suggesting that clinical scheduling strategies should be dynamically adjusted according to employees’ work tenure in future practice.

### 6.6. The Shiny Web Calculator and Open‐Source Code

We developed an interactive Shiny web calculator based on the optimal random forest model, accessible at https://dinyou.shinyapps.io/Nurse_Burnout_RiskCalc/. The full open‐source code, deployment documentation, software dependencies, and version‐controlled environment (using renv) are available on GitHub: https://github.com/dingyou123/Identifying-Key-Predictors-and-Developing-a-Machine-Learning-Model-for-Nurse-Burnout-in-China. The repository includes a formal stability statement (the tool will be maintained for at least 5 years post‐publication) and evidence of cross‐environment validation (tested on Windows, macOS, Linux, iOS, Android, and major browsers with consistent functionality and outputs).

## 7. Discussion

### 7.1. Comparison of Machine Learning Model Performance

This study compared seven machine learning models for predicting nurse job burnout. All models were trained on the 80% training set and validated on the 20% independent test set. Random forest achieved the highest training AUC (0.991), PR‐AUC (0.997), accuracy (0.949), and F1‐score (0.965). On the test set, random forest had the highest AUC (0.879)—tied with LightGBM—as well as the highest accuracy (0.837), sensitivity (0.870), F1‐score (0.890), and NPV (0.643). The test set PR‐AUC of random forest was 0.940, which is higher than the baseline prevalence (75.7%), reflecting model robustness to class imbalance and reliable identification of the minority class (nonburnout nurses) [27].

Our primary success criterion for model comparison was sensitivity because early identification of nurses at risk of burnout is critical. Missing a true burnout case (false negative) would delay intervention and potentially worsen outcomes. In contrast, a false positive (labeling a nonburnout nurse as at risk) incurs only a low‐cost secondary assessment (e.g., a brief interview) with no harm. Based on the test set results, random forest was selected as the optimal prediction model for nurse burnout. Its calibration was assessed using the HL test (*p* = 0.3505) and Spiegelhalter test (*p* = 0.1735), with a Brier score of 0.1052; the calibration curve is presented in Figure 4 [28]. DCA (Figure 5) showed that across a wide range of threshold probabilities, random forest provided higher net benefit than both “treat‐all” and “treat‐none” strategies [29].

The advantages of random forest over traditional logistic regression are as follows [30, 31].

On the test set, random forest outperformed logistic regression in sensitivity (0.870 vs. 0.690), accuracy (0.837 vs. 0.718), F1‐score (0.890 vs. 0.787), and NPV (0.643 vs. 0.453). Its PR‐AUC (0.940) was substantially higher than the baseline prevalence (75.7%), demonstrating strong capability for handling class imbalance.

Compared with logistic regression, random forest offers several advantages: (1) automatic capture of nonlinear relationships without prespecified forms; (2) random forest can capture feature interactions directly without requiring prespecified terms, whereas logistic regression would need manually constructed interaction variables to account for such effects; (3) robustness to multicollinearity (e.g., between work duration and age group); and (4) inherent strength for imbalanced data via cost‐sensitive learning (specificity: 0.731, NPV: 0.643).

### 7.2. Risk Stratification and Clinical Utility

This study employed a random forest model to integrate multidimensional burnout‐related factors, using the model’s predicted probability as an individualized composite risk index. The optimal threshold for this composite index was determined to be 0.527 via the maximum Youden index method [32, 33]. Serving as a decision node that captures the synergistic action of multidimensional factors, this threshold maximally distinguishes nurses with burnout from those without. Based on this threshold and half its value (0.264), three clinical risk grades were defined: low risk (< 0.264), moderate risk (0.264–0.527), and high risk (> 0.527).

The probability density distribution plot (Figure 6) clearly revealed the intrinsic association between different risk tiers and the distribution of burnout‐related factors. The predicted scores of nurses with actual burnout exhibited a marked right‐skewed distribution, heavily concentrated to the right of the high‐risk threshold, indicating that this group was generally exposed to extremely unfavorable levels across multiple interpersonal, health‐behavioral, and organizational factors. In contrast, the scores of nurses without burnout clustered tightly to the left of the low‐risk threshold, reflecting the stable presence of protective factors. The overlapping area of the two density curves within the moderate‐risk interval precisely captured the intermediate population with some factors at critical levels and volatile risk, thereby confirming the rationality of this threshold‐based stratification in reflecting the cumulative effects of the factors. The dual‐axis plot (Figure 7) further substantiated the discriminative power of this stratification through a stepwise escalation in actual burnout rates: the burnout rates in the low‐, moderate‐, and high‐risk groups were 12.8%, 56.2%, and 93.7%, respectively, with the high‐risk group capturing 84.8% of true burnout cases. As shown in the Sankey diagram (Figure 8) and Table 4, among 210 nurses with confirmed burnout, 178 (84.8%) were accurately anchored into the high‐risk group, and only 5 (2.4%) fell into the low‐risk group; among the 67 nurses without burnout, more than half (50.7%) were correctly retained in the low‐risk group. Overall, the model channeled 97.6% of true burnout cases into the moderate‐ and high‐risk intervention groups while avoiding excessive intervention for the majority of nonburnout nurses, demonstrating the precision in capturing high‐risk individuals and the acceptable specificity achieved by this threshold‐based stratification strategy after integrating information from the relevant factors.

The above results robustly demonstrate that the optimized threshold of 0.527 is not an isolated probability cut point, but rather a highly condensed representation of the synergistic effects of multiple burnout‐related factors. The high‐risk tier essentially delineates the subgroup of nurses simultaneously exposed to adverse conditions across multiple dimensions—such as collegial relationships, nurse–physician collaboration, exercise frequency, night shift burden, and training/examination demands—while the moderate‐risk tier mainly encompasses a transitional population for whom some factors have already raised warning signals but several buffering resources remain. The smooth convergence of the learning curve (Figure 9) further confirms that the model has adequately learned the complex patterns among these factors from the current sample (*n* = 1391), with no evident underfitting. Therefore, the current risk stratification tool based on the composite probability threshold has successfully translated the correlations of multidimensional factors into clinically actionable decision‐making evidence, capable of robustly supporting a tiered, proactive burnout prevention system. Future external validation will further enhance the robustness and generalizability of this tool across diverse healthcare settings.

### 7.3. Factors Associated With Nurse Job Burnout (Exploratory Analysis)

Note: Due to the cross‐sectional study design, causal relationships cannot be established. The following analyses are exploratory in nature and only describe associations observed in the data.

This study employed SHAP analysis to deeply interpret the prediction mechanism of the random forest model, systematically revealing the multidimensional factor structure underlying nurse burnout and their interaction effects [1, 34, 35]. The analysis indicates that burnout is characterized by not any single factor but rather reflects a risk network woven together by interpersonal resources, personal health assets and professional resilience, and organizational management practices. Among the top ten features by importance, the quality of workplace interpersonal relationships was once again identified as the strongest protective‐associated factor: colleague relationship ranked highest with an importance score of 0.178, closely followed by work duration (0.090) and weekly exercise frequency (0.033), forming the core protective factor cluster; family support, monthly income, monthly training frequency, education level, only‐child status, hospital level, and weekly night shift hours collectively accounted for the remaining explanatory space of risk.

Among these factors, colleague relationship is not only the most important independent feature but also the hub within the interaction network [36, 37]. The SHAP waterfall plot and individual force plots intuitively demonstrated that harmonious colleague relationships and longer work duration are associated with lower predicted risk, shifting the overall mean prediction from 0.80 down to 0.58. In the extreme high‐risk case (predicted probability: 0.993), poor colleague relationship and very short work duration (only 2 years) showed positive SHAP values of +0.0509 and + 0.0425, respectively; in the low‐risk case (predicted probability 0.001), 35 years of work duration and highly harmonious colleague relationships showed strongly negative SHAP values of −0.360 and −0.285. This finding aligns closely with the job demands‐resources (JD‐R) model, which suggests that supportive social relationships may offset the depletion caused by job demands [38]. Friedman’s H‐statistic further revealed that colleague relationship exhibited a strong interaction intensity of 0.330 and formed close linkages with hospital level (*H* = 0.244) and family support (*H* = 0.233), suggesting that the protective association of relationship quality may be further strengthened within a supportive organizational hierarchy and with family emotional backing. Therefore, the focus of intervention should not stop at individual emotional consolation but should establish a systemic relationship‐cultivation mechanism built on team building, buddy systems, and anonymous feedback.

Personal health assets and professional resilience likewise constitute an indispensable line of defense. Work duration and exercise frequency ranked second and third in importance, respectively, and their nonlinear interactive effects deserve particular attention. The SHAP dependence plots showed that when work duration is short, prolonged night shifts are associated with markedly elevated burnout risk; however, once experience exceeds approximately 20 years, the predicted curve stabilizes in the negative range regardless of night shift burden, suggesting a pattern consistent with an “immune barrier” among senior nurses [39]. The protective association of exercise frequency with burnout risk was more pronounced among non–only‐child nurses, implying that nurses from different social backgrounds may show different associations with the same health behavior. These findings suggest potential directions for occupational health promotion, such as setting a cap on night shifts and mandatory rest days for junior nurses, establishing a tenure‐matched flexible scheduling system, and providing fitness subsidies and group sports activities [40–42].

Although organizational management factors ranked relatively lower in univariate importance, their institutional effects are associated with burnout risk through a complex interaction network. Monthly training frequency and weekly night shift hours represent persistent institutional loads, and the SHAP dependence plots further suggested a notable synergistic association between education and income: high education combined with high income showed the lowest SHAP values. Similarly, nonlinear positive synergies were observed between family support and colleague relationships and between training frequency and income. These findings suggest potential areas for optimizing organizational management, such as adjusting the frequency of training assessments, quantifying night shift intensity into salary coefficients, and organizing family support activities [43, 44].

In summary, the risk landscape delineated by the random forest model coupled with the SHAP interpreter provides a systematic exploratory framework for understanding factors associated with nurse burnout—from single‐factor identification to interaction‐based patterns. Given the cross‐sectional design, these findings should be interpreted as hypothesis‐generating rather than causal. Future longitudinal studies are needed to test the predictive directionality of the observed associations. At a time of escalating public healthcare demands and persistent challenges to nursing workforce stability, scientific risk stratification must be embedded into institutional decision‐making, enabling peer relationship cultivation, experience‐based scheduling protection, differentiated health promotion, and human resource policies that respect professional value to together constitute a resilient ecosystem for occupational health protection.

### 7.4. Analysis of the Clinical Application of the Prediction Model

Current predictive model research often suffers from insufficient translational application, with most studies failing to develop operational tools suitable for clinical practice. Existing research primarily employs two distinct approaches to visualization, each with notable differences in application characteristics. In models constructed using traditional statistical methods, nomograms serve as the primary visualization tool [12]. Although such static visualization tools can intuitively display variable weights, they exhibit significant limitations: model parameters become fixed and cannot be dynamically updated, data adjustments require replotting, and they lack remote collaboration and real‐time interaction capabilities [44].

In contrast, studies based on machine learning algorithms place greater emphasis on operational applicability. This study developed a web application based on the Shiny framework, featuring a two‐level functional interface: a numerical input interface supporting the entry of 12 indicators, and a result display interface that visually presents individualized prediction probabilities and the contribution degree of each variable. The application demonstrates good operability, interactivity, and visibility. Furthermore, the webpage is accessible to all users, providing convenience for nursing managers and promoting the practical deployment and application of the constructed prediction model.

### 7.5. Conclusion

This study developed and compared seven machine learning models to predict job burnout among 1391 Chinese nurses. The random forest model performed best on the test set (AUC = 0.879, PR‐AUC = 0.940) and reliably identified high‐risk individuals. Key associated features included colleague relationship, work duration, exercise frequency, night shift hours, and training frequency, with several synergistic interactions. The three‐tier risk stratification (low, moderate, and high) based on the optimal cutoff (0.527) correctly assigned 97.6% of true burnout cases to the moderate‐ or high‐risk groups. The model showed good calibration (HL test *p* = 0.3505), and DCA indicated net benefit superior to “treat‐all” or “treat‐none” strategies. In addition, an interactive Shiny web calculator based on the random forest model was developed to facilitate individual self‐assessment and manager‐led screening. Future external validation studies using multicenter datasets from different geographic regions and hospital types are needed to confirm the generalizability of our findings. Pending such validation, the model and the online tool may serve as practical references for early identification and stratified intervention in nursing management.

## 8. Limitations

Several limitations should be acknowledged. First, model evaluation relied solely on internal cross‐validation and has not yet been externally validated in independent datasets. This may limit the external validity (generalizability) of our findings to other populations, hospital settings, or geographic regions. Second, the sample size was relatively limited, and although we addressed class imbalance algorithmically, the sample was derived from a convenience sample which may not fully represent the broader population of Chinese nurses. Third, the absence of an external validation cohort means that the reported AUC and calibration performance may be optimistic. Future studies with larger, multicenter samples and additional objective indicators (e.g., electronic health record data and objective workload metrics) are needed to perform rigorous external validation and further enhance generalizability and robustness.

## 9. Implications and Recommendations for Practice

It is recommended to integrate the prediction model into hospital human resource or nursing information systems for automated burnout risk assessment and reporting. This would support data‐driven decision‐making, enabling timely targeted interventions for high‐risk nurses, thereby facilitating dynamic monitoring of nurse well‐being and improving nursing management efficiency.

## Author Contributions

Conceptualization, Xiao Gan; formal analysis, Zirong Li; funding acquisition, Xiao Gan and Yanping Ying; methodology, Zirong Li; data collection, Qinghua Fan; software, Zirong Li; visualization, Zirong Li; writing–original draft, Zirong Li; writing–review and editing, Yuhui Chen and Jinbi Wei; and critical revision and final approval, all authors.

## Funding

This work was supported by the First Affiliated Hospital of Guangxi Medical University Nursing Clinical Research Climbing Program Angel Star Project (YYZS2021007) and Nursing Clinical Research Climbing Project of the First Affiliated Hospital of Guangxi Medical University (YYZS2020025).

## Ethics Statement

This study has passed the ethical review of the First Affiliated Hospital of Guangxi Medical University, and the ethical review number is 2025‐E0820.

## Consent

This study obtained approval for an exemption of participant signed informed consent.

## Conflicts of Interest

The authors declare no conflicts of interest.

## Supporting Information

Additional supporting information can be found online in the Supporting Information section.

## Supporting information


**Supporting Information** This manuscript is accompanied by the following supporting information: File Name: TRIPOD + AI Statement.pdf. Description: This file contains the completed TRIPOD + AI (Transparent Reporting of a multivariable prediction model for Individual Prognosis Or Diagnosis + Artificial Intelligence) checklist, which provides a detailed overview of the reporting items for our clinical prediction model study.

## Data Availability

The data that support the findings of this study are available from the corresponding author upon reasonable request.
