# Supplementary figures and images for: Identifying Key Predictors and Developing a Machine Learning Model for Nurse Burnout in China
Source: J Nurs Manag. 2026 Jul 13;2026:8179894. doi: 10.1155/jonm/8179894 (PMC13364991; doi:10.1155/jonm/8179894)

# AdaBoost Hyperparameter Tuning

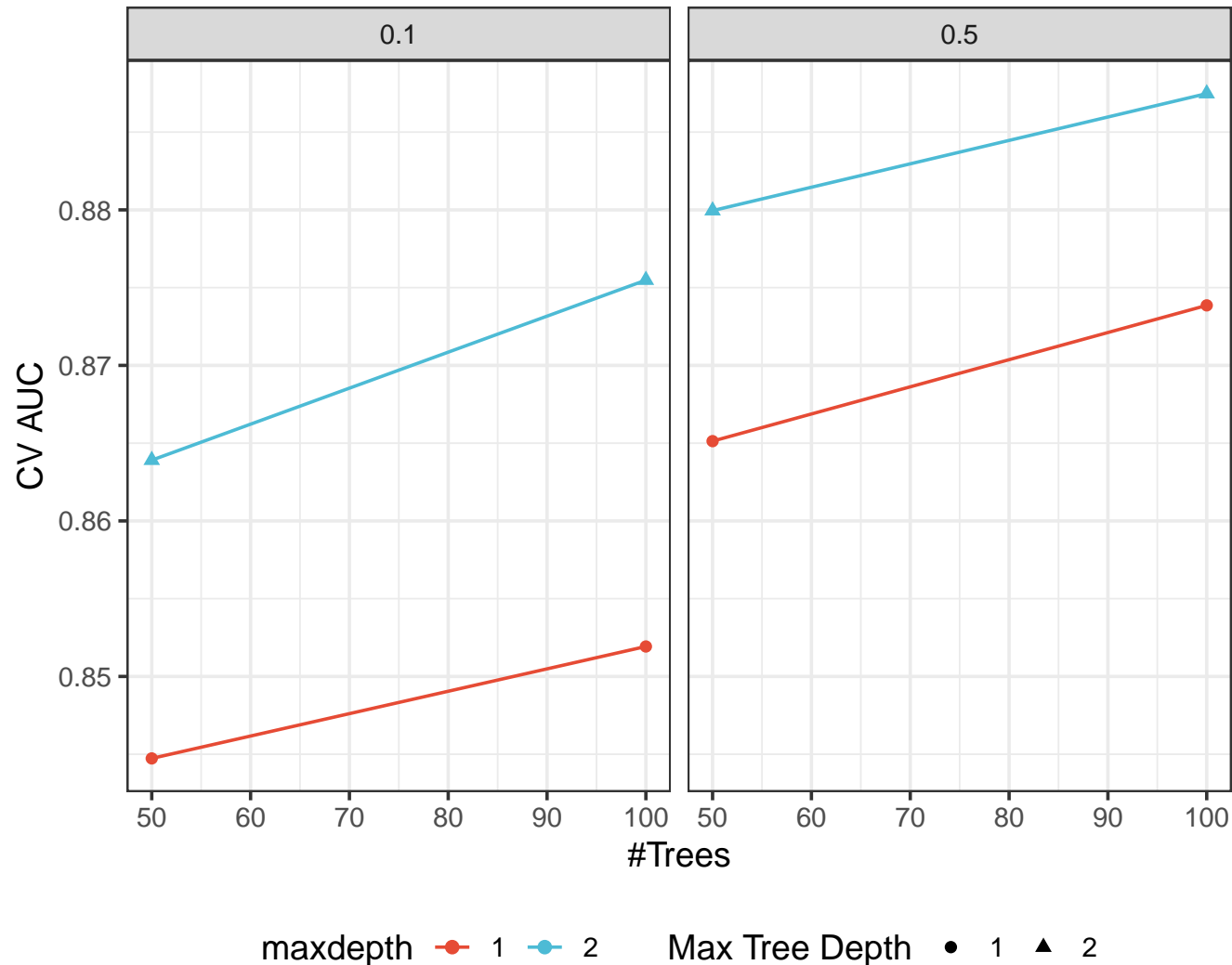

Supplement: Supplementary file 1 — Supporting Information This manuscript is accompanied by the following supporting information: File Name: TRIPOD + AI Statement.pdf. Description: This file contains the completed TRIPOD + AI (Transparent Reporting of a multivariable prediction model for Individual Prognosis Or Diagnosis + Artificial Intelligence) checklist, which provides a detailed overview of the reporting items for our clinical prediction model study. [file JONM-2026-8179894-s001.zip › AdaBoost_Tuning_Plot.pdf]

# ElasticNet Hyperparameter Tuning

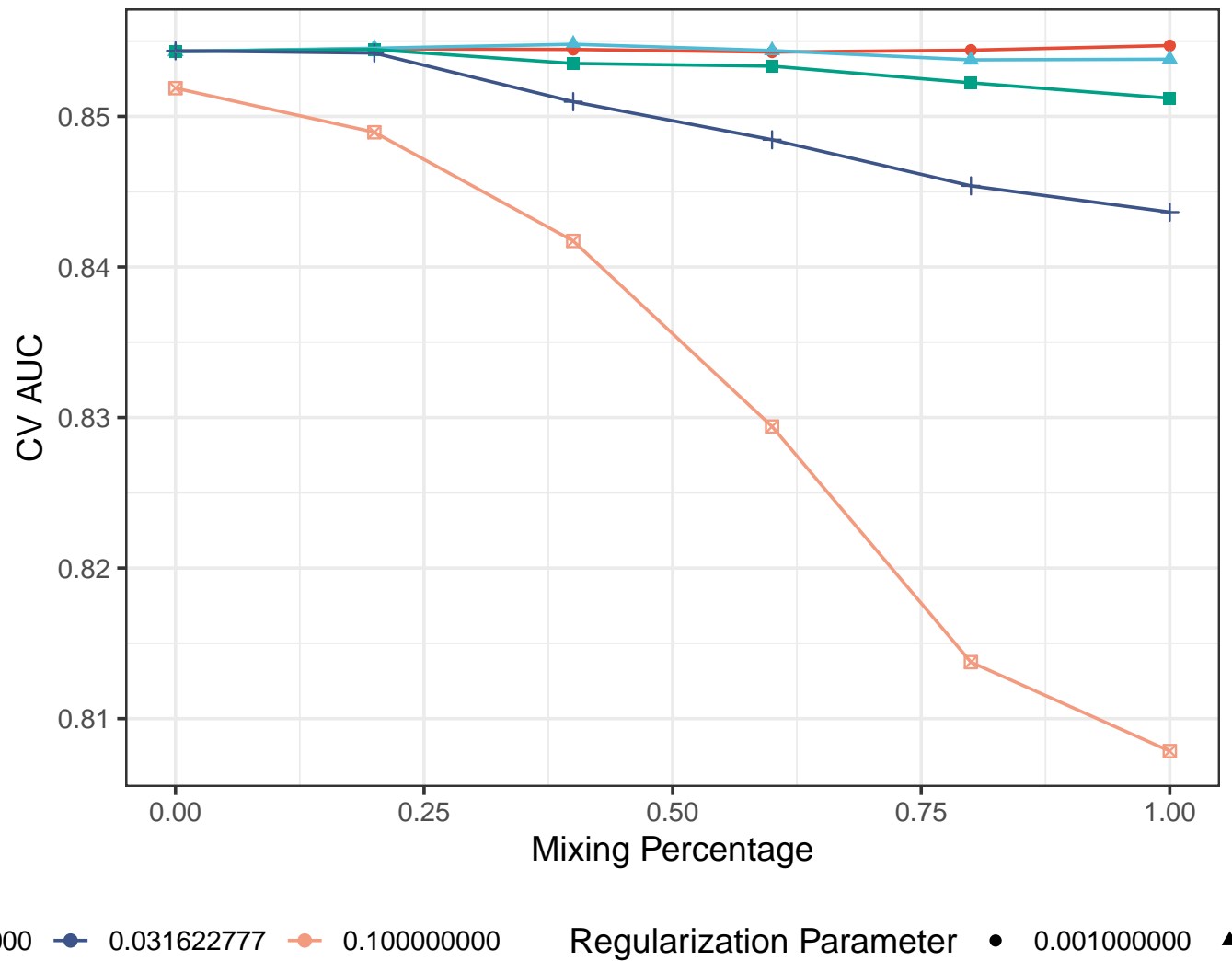

Supplement: Supplementary file 1 — Supporting Information This manuscript is accompanied by the following supporting information: File Name: TRIPOD + AI Statement.pdf. Description: This file contains the completed TRIPOD + AI (Transparent Reporting of a multivariable prediction model for Individual Prognosis Or Diagnosis + Artificial Intelligence) checklist, which provides a detailed overview of the reporting items for our clinical prediction model study. [file JONM-2026-8179894-s001.zip › ElasticNet_Tuning_Plot.pdf]

# KNN Hyperparameter Tuning

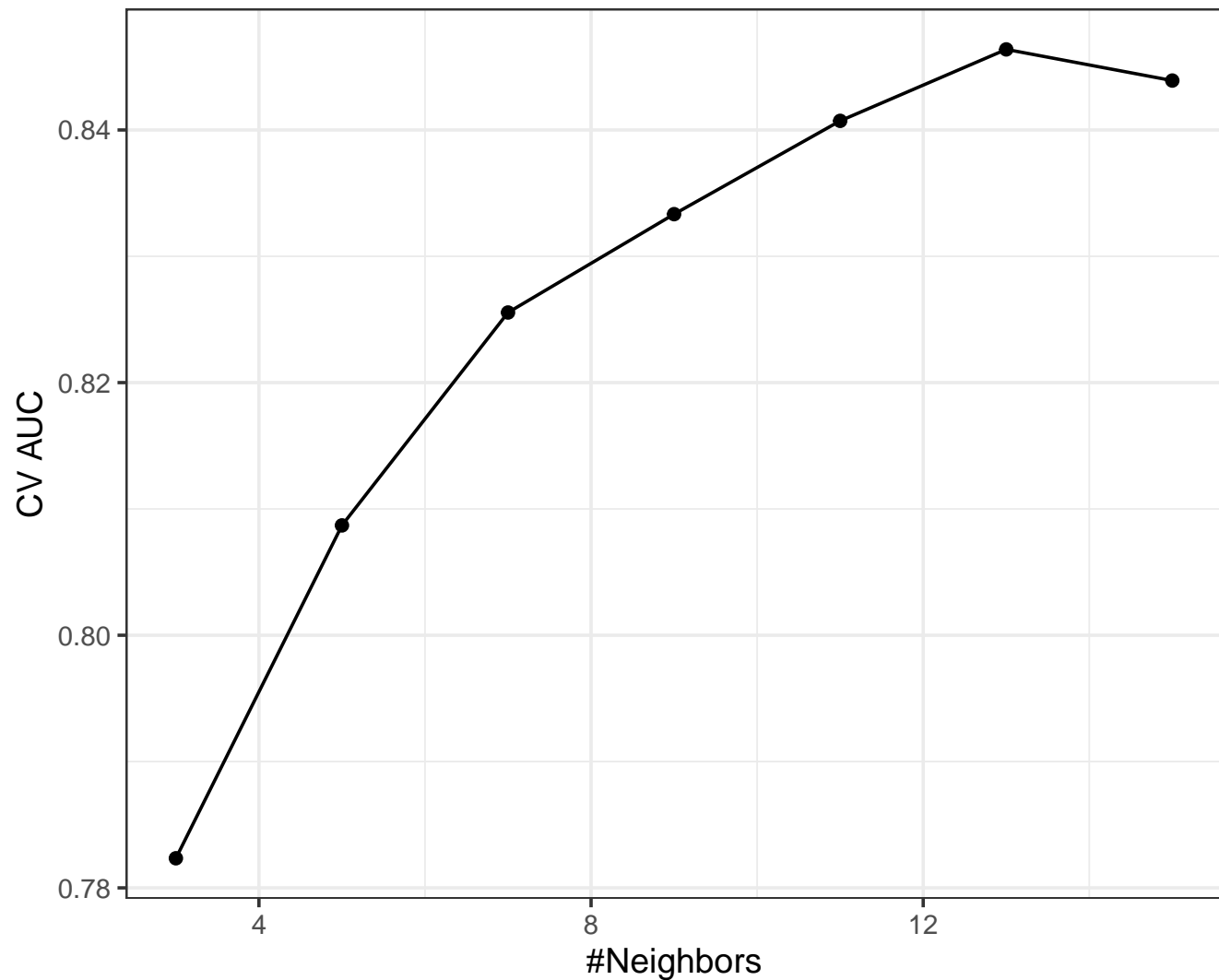

Supplement: Supplementary file 1 — Supporting Information This manuscript is accompanied by the following supporting information: File Name: TRIPOD + AI Statement.pdf. Description: This file contains the completed TRIPOD + AI (Transparent Reporting of a multivariable prediction model for Individual Prognosis Or Diagnosis + Artificial Intelligence) checklist, which provides a detailed overview of the reporting items for our clinical prediction model study. [file JONM-2026-8179894-s001.zip › KNN_Tuning_Plot.pdf]

# LightGBM Bayesian Optimization History

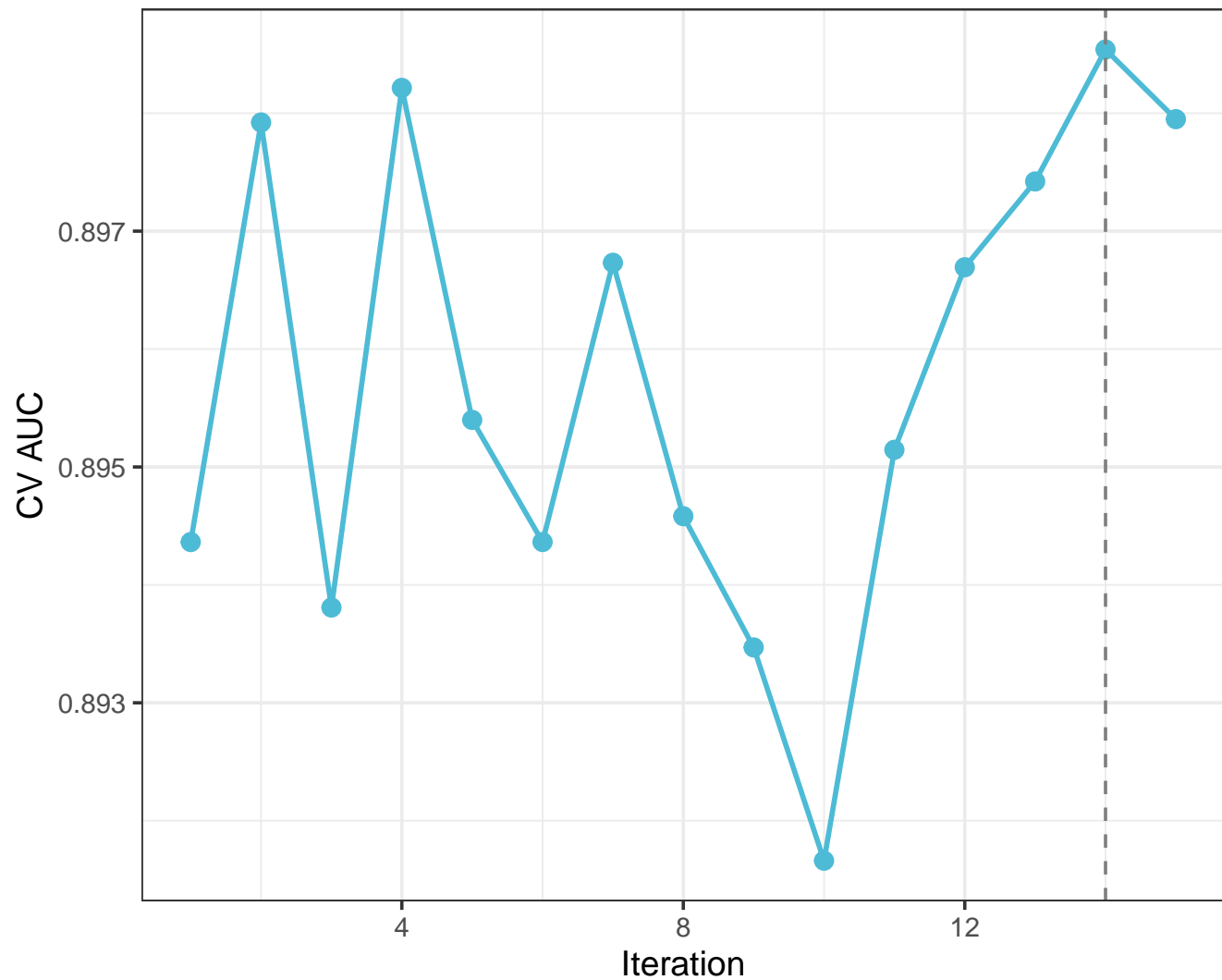

Supplement: Supplementary file 1 — Supporting Information This manuscript is accompanied by the following supporting information: File Name: TRIPOD + AI Statement.pdf. Description: This file contains the completed TRIPOD + AI (Transparent Reporting of a multivariable prediction model for Individual Prognosis Or Diagnosis + Artificial Intelligence) checklist, which provides a detailed overview of the reporting items for our clinical prediction model study. [file JONM-2026-8179894-s001.zip › LightGBM_Tuning_Plot.pdf]

# RandomForest Hyperparameter Tuning

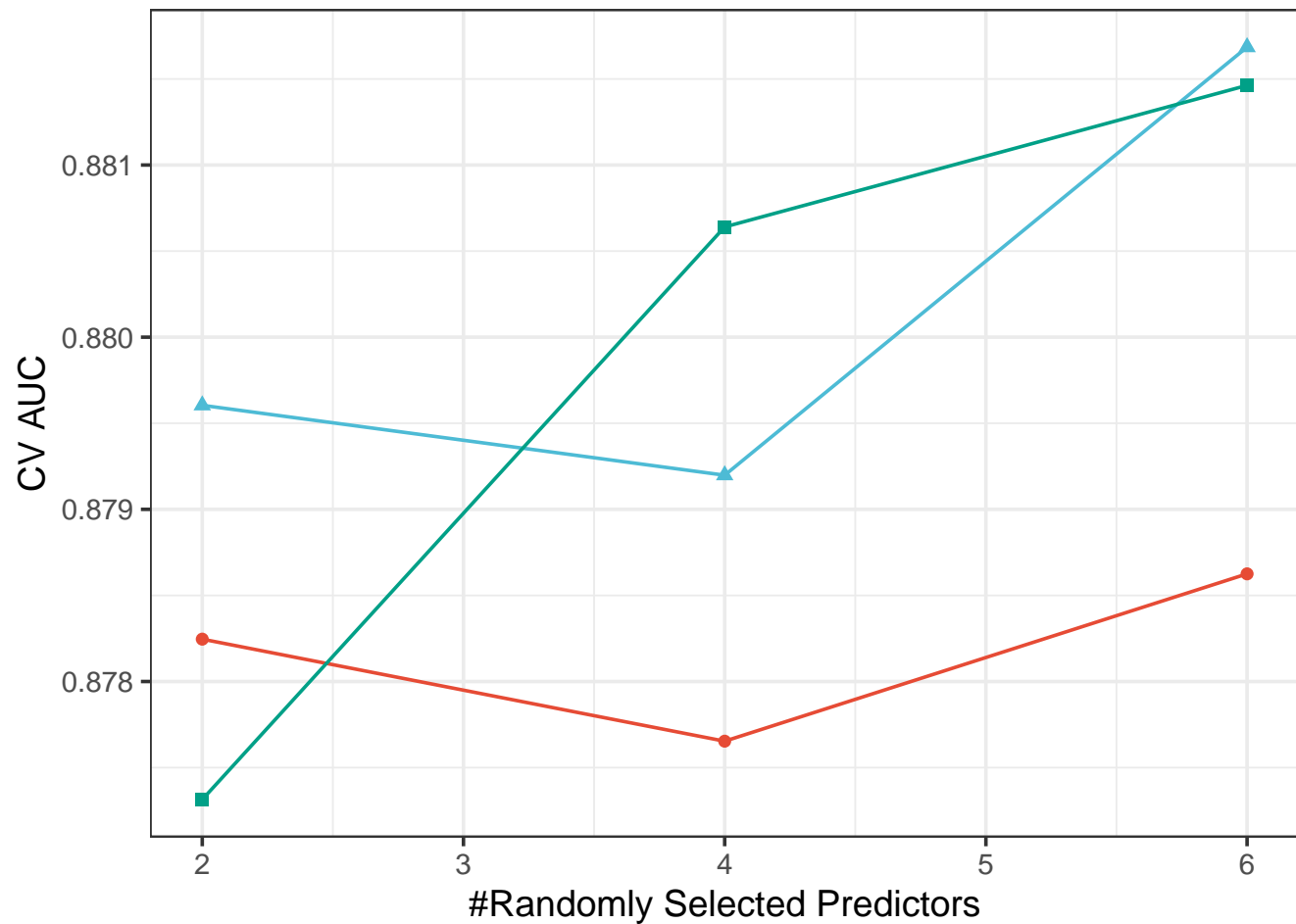

min.node.size    ● 5    ▲ 10    ■ 15    Minimal Node Size    ● 5    ▲ 10    ■ 15

Supplement: Supplementary file 1 — Supporting Information This manuscript is accompanied by the following supporting information: File Name: TRIPOD + AI Statement.pdf. Description: This file contains the completed TRIPOD + AI (Transparent Reporting of a multivariable prediction model for Individual Prognosis Or Diagnosis + Artificial Intelligence) checklist, which provides a detailed overview of the reporting items for our clinical prediction model study. [file JONM-2026-8179894-s001.zip › RandomForest_Tuning_Plot.pdf]

# XGBoost Bayesian Optimization History

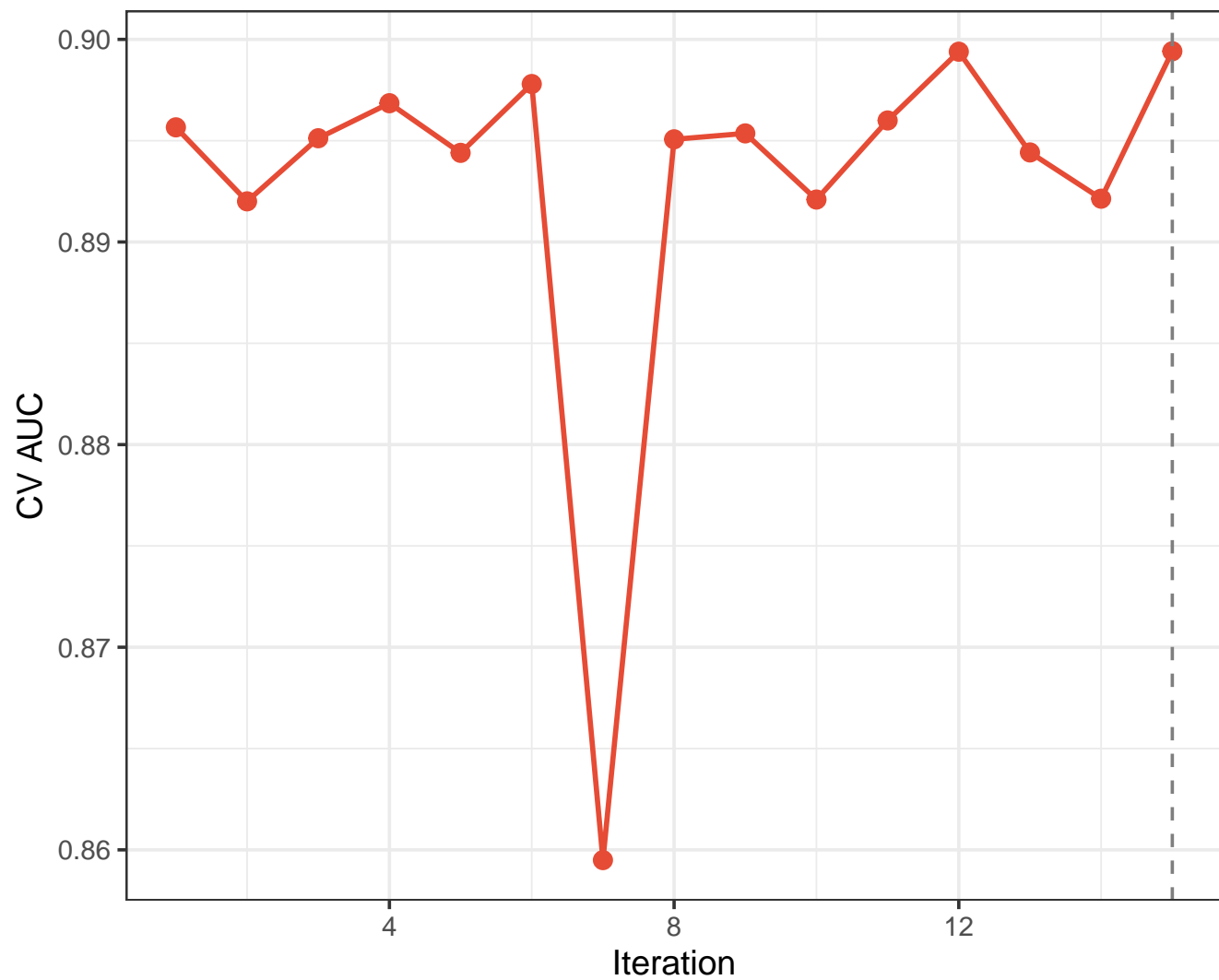

Supplement: Supplementary file 1 — Supporting Information This manuscript is accompanied by the following supporting information: File Name: TRIPOD + AI Statement.pdf. Description: This file contains the completed TRIPOD + AI (Transparent Reporting of a multivariable prediction model for Individual Prognosis Or Diagnosis + Artificial Intelligence) checklist, which provides a detailed overview of the reporting items for our clinical prediction model study. [file JONM-2026-8179894-s001.zip › XGBoost_Tuning_Plot.pdf]
